# How to shore up trust during the “cold-period” between pandemics – closing the public trust gap in pandemic preparedness frameworks

**DOI:** 10.1080/16549716.2026.2662800

**Published:** 2026-04-23

**Authors:** Kimon Papadopoulos, Viktor von Wyl, Felix Gille

**Affiliations:** a Digital Society Initiative (DSI), University of Zürich, Zurich, Switzerland; b Institute for Implementation Science in Health Care (IfIS), University of Zürich, Zurich, Switzerland

**Keywords:** Public trust, pandemic preparedness, World Health Organization, health policy, public health

## Abstract

**Background:**

Public trust is crucial for effective pandemic response, influencing compliance and cooperation. Past pandemics have revealed trust gaps that have weakened response efforts. However, there is limited evidence-based guidance on how to integrate public trust into preparedness frameworks.

**Objective:**

The objective of this study is to provide guidance on embedding public trust-building principles in pandemic preparedness frameworks and strategies.

**Methods:**

A comparative analysis of the WHO’s ‘five C’ framework and a public trust in health systems framework was conducted to assess the integration of trust-building principles into the WHO’s framework. Each trust-building principle was classified as explicitly present, implicitly present, or absent. Absent trust principles highlight areas for strengthening public trust in pandemic preparedness. A quality checklist, adapted from research on European health data-sharing legislation, ensured systematic evaluation.

**Results:**

The analysis of the five C framework highlights varying integration of trust principles in pandemic preparedness. ‘Community Protection’ has strong trust coverage, while ‘Collaborative Surveillance’, ‘Safe and Scalable Care’, ‘Access to Countermeasures’, and ‘Emergency Coordination’ present gaps in public trust promotion, particularly in information quality, familiarity, privacy, anonymity, and time. Time is frequently absent, suggesting a need for greater emphasis.

**Conclusions:**

Governments and other health policy stakeholders should prioritize incorporating familiarity, information quality, privacy, autonomy, time, and anonymity into appropriate aspects of pandemic preparedness frameworks. Incorporating these trust principles during inter-pandemic periods will assist in the promotion and strengthening of public trust in pandemic preparedness measures, ensuring effective public support and deference during a public health crisis response.

## Paper Context

- **Main findings:** The World Health Organization’s five C framework partially integrates public trust-building principles, yet has gaps in the principles of familiarity, privacy, anonymity, information quality, and certain aspects of time.
- **Added knowledge:** This study identifies specific trust-related strengths and deficiencies in an international pandemic preparedness framework and provides evidence-based recommendations for improvement.
- **Global health impact for policy and action:** Strengthening public trust by incorporating key trust-building principles into pandemic preparedness frameworks will enhance public compliance and cooperation in actionable pandemic mitigation measures during future public health crises.

## Background

Preparedness frameworks have been a major component of global and public health for decades. Preparedness frameworks are an instrumental tool in creating a resilient infrastructure toward responding to infectious disease epidemics and pandemics appropriately and effectively [1,2]. Preparedness, as defined by the United Nations and the World Health Organization, is the ability of governments, organizations, communities, and individuals to anticipate, detect, respond to, and recover from health emergencies. It involves establishing systems to assess risks and rapidly mobilize resources during crises [1]. In modern public health, the outbreaks of severe acute respiratory syndrome (SARS), avian influenza, and swine influenza in the 2000s have played a pivotal role in shaping early 21st-century approaches to pandemic preparedness [2,3]. Now, in the aftermath of the COVID-19 pandemic, new pandemic preparedness frameworks have been developed or are being developed at the national, regional, and international levels of governance. These include models by the US and European Centers for Disease Control, as well as the World Health Organization, designed to prepare for the next pandemic by utilizing lessons learned from COVID-19 [4–6]. These frameworks help guide governments put infrastructures, systematic processes, legal bases, strategic partnerships, and other actionable operative recommendations in place in order to be better prepared for future pandemics [7]. However, frameworks have little impact if they are not well accepted and implemented appropriately at the population level; and this cannot be accomplished without public trust [8].

At its core, public trust can be best defined as the expectation that institutions, organizations, and individuals who manage, regulate, or deliver services to the public will prioritize the public’s best interests and demonstrate integrity when doing so [9]. In pandemic response, the importance of public trust is two-fold and reciprocal. The public must trust that the health and political stakeholders charged with safeguarding their health are acting within the best interests of the public; and those same health authorities must trust the public to participate in their health interventions and take collective action [8,10,11]. When these conditions are met, health authorities have a surplus of ‘social-trust capital’ to utilize in order to get significant portions of the public to respond to and participate in public health outbreak control and mitigation measures [10]. During the COVID-19 pandemic, issues arose regarding trust in public health authorities, which negatively affected public participation in crucial health interventions, such as vaccine uptake, demonstrating gaps in the trust of pandemic response measures. Consequently, these gaps must be addressed in order to better combat the next pandemic, especially as new infectious disease outbreaks continue to loom, including recent instances of monkeypox, norovirus, and H5N1 avian flu [12–20]. Therefore, we must ask, how can trust be strengthened during a so-called ‘cold-period’ between pandemics?

Although assessing and fostering public trust is a complex and time-intensive process, it is essential to prioritize this effort well before the next pandemic. However, there is a lack of evidence-based guidance on incorporating or implementing trust in pandemic preparedness in preparation of this study, with only one article found discussing strategies on how to incorporate trust into pandemic preparedness, ‘A practical agenda for incorporating trust into pandemic preparedness and response’ by Bollyky and Petersen [21]. Therefore, the objective of this paper is to provide guidance on incorporating public trust-building principles into pandemic preparedness frameworks in order to strengthen public trust in public health interventions before the next pandemic. This will be accomplished with a comparative framework analysis between the World Health Organization’s ‘five C’ framework on health emergency prevention, preparedness, response, and resilience, and a framework detailing how to promote public trust in the health system [4,8]. In the following section, the rationale behind the choice of frameworks for this study, along with brief introductions to said frameworks, will be provided.

### The five Cs of health emergency prevention, preparedness, response, and resilience

Although other contemporary preparedness frameworks exist, such as those developed by the US and European Centers for Disease Control, the World Health Organization’s framework was selected as the most suitable reference point for this study [4–6]. Unlike national or regional frameworks, it was designed for cross-national use and explicitly links global, national, and local levels of preparedness. As well, its structure incorporates governance, operational, and community domains, making it especially well-suited for systematic comparison [4]. In addition, the World Health Organization’s framework directly informs pandemic planning and guidance in many member states, ensuring that findings from a comparative framework analysis are broadly relevant in both global and national contexts [4]. These attributes align with the aims of this study’s comparative framework analysis, making the five C framework the most methodologically appropriate choice.

The five C framework was developed by the World Health Organization in the aftermath of the COVID-19 pandemic and incorporates lessons learned from that pandemic to help guide countries in strengthening their response and prevention measures in the face of future public health emergencies (PHEs), such as a pandemic [4,22]. To manage the potential increasing complexity and scale of PHEs, the five Cs represent an interlinked system approach to encompass and complement existing PHE response pillars, include multi-stakeholder and multidisciplinary approaches, and align with existing One Health and zoonotic disease prevention strategies [4]. The five Cs are as follows: collaborative surveillance, community protection, safe and scalable care, access to countermeasures, and emergency coordination. These can be seen in more detail in Table 1 [4].

**Table 1.** World Health Organization’s five C framework (adapted from ‘strengthening the global architecture for Health emergency prevention, preparedness, response and resilience’).

The Five Cs of Health Emergency, Prevention, Preparedness, Response, and Resilience	
Collaborative Surveillance	Building capacity and fostering collaboration among a wide range of stakeholders, both inside and outside the health sector, to enhance public health intelligence and strengthen evidence-based decision-making.
Community Protection	Creating community-centered initiatives aimed at safeguarding the health and well-being of affected individuals, such as vaccination, to prevent infectious diseases.
Safe & Scalable Care	Equipping health systems to be ready to respond swiftly to emergencies, ensuring communities have access to high-quality healthcare in safe and functional environments both during and after crises.
Access to Countermeasures	Successful testing, treatment, and protection of communities during health emergencies rely on timely, adequate, and equitable access to medical countermeasures, including diagnostics, therapeutics, vaccines, medical devices, and equipment.
Emergency Coordination	Effective coordination of health emergency preparedness and response systems is essential for systematically mobilizing and deploying the necessary resources (including knowledge, data, finances, materials, technical expertise, and operations) to prepare for, prevent, detect, alert, and respond swiftly to any health emergency.

### Public trust-building principles in the health system framework

The public trust in the health system framework was selected to assess the integration of trust principles in the five C framework due to its comprehensive overview of what is conducive to the establishment of trust in the overall health system, which includes pandemic preparedness [8]. As well its transferability has been successfully proven in other case studies [8,23,24].

The public trust in the health system framework (Table 2) was created in the context of the National Health Service (NHS) in England [8]. This framework comprises thirteen causal themes, which detail and define the factors that increase public trust in the health system, providing insights for both policymaking and public trust measurement [8,25]. This framework was developed utilizing a review of previous frameworks of public trust in the healthcare system and trust theory, psychometric principles, and an analysis of three qualitative NHS England case studies [25].

**Table 2.** Public trust in the health system framework (adapted from ‘themes building public trust in the health system’).

Themes	Explanations of Terms in Relation to Public Trust
*Familiarity*	Previous positive experiences with an actor or institution enhances a sense of familiarity, leading to higher public trust.
*Active regulatory systems*	The presence of functioning and transparent regulatory mechanisms provides oversight and accountability, leading to higher public trust.
*Anonymity*	Ensuring that personal data is anonymized prior to sharing reduces concerns over identification, leading to higher public trust.
*Autonomy*	Respecting and supporting an individual’s choices strengthens their confidence in participation, leading to higher public trust.
*Gut feeling*	When people feel something is trustworthy on instinct, it can shape their willingness to trust, leading to higher public trust.
*Information quality*	Providing information that is accurate, honest, and transparent supports informed decision-making, leading to higher public trust.
*Privacy*	Maintaining the confidentiality of personal information protects an individuals right to privacy, leading to higher public trust.
*Potential*	A perceived ability of an actor or system to achieve intended outcomes fosters confidence in their success, leading to higher public trust.
*Respect*	Demonstrating mutual respect between public stakeholders and institutional actors encourages constructive engagement, leading to higher public trust.
*Security*	When systems and processes are perceived as secure and reliable, confidence in their safety increases, leading to higher public trust.
*Certainty about the future*	Efforts to anticipate future risks and proactively plan responses create a sense of preparedness, leading to higher public trust.
*Net benefit*	Perceived benefits, whether individual, societal, or systemic, justifies participation and/or support, leading to higher public trust.
*Time*	Allowing adequate time for processes and decisions to unfold signals thoroughness and care, leading to higher public trust.

## Methods

For this study, a comparative analysis was conducted between the World Health Organization’s five C framework and the public trust in the health system framework developed by FG in order to identify alignments and gaps between the two frameworks in the context of trust-building principles [4,8,26]. The objective of this analysis was to determine if and how public trust principles are integrated into the World Health Organization’s framework and to identify potential gaps where public trust-building measures could be strengthened. A comparative approach was particularly well-suited to this objective, as it enabled a structured, side-by-side assessment of two established frameworks. By directly comparing a trust-specific framework with a preparedness-specific framework, the analysis highlights concrete gaps where trust-building principles could be better integrated, thereby aligning the method with the study’s objective of providing evidence-based recommendations for strengthening public trust in preparedness strategies [21,27].

The two frameworks were compared and integrated by analyzing which public trust-building principles from the public trust in the health system framework were represented within each ‘C’ of the five C framework, either explicitly or implicitly, while also identifying which trust-building principles were absent from each ‘C’. Not all trust-building principles from the public trust in the healthcare system were expected to be included in the five C framework. The principle of *gut feeling* was excluded as it was not found to be directly relevant to the preparedness contexts of the five Cs and their subpoints. *Gut feeling*, which relates to instinctive or emotional reasonings for trust, may influence health behaviors, but it cannot be systematically incorporated into preparedness planning. Preparedness frameworks are designed around measurable, structural, and procedural capacities, whereas *gut feeling* reflects emotional responses that are often unpredictable and not always logical [4,8]. For example, during the COVID-19 pandemic, some individuals reported trusting vaccines simply because it ‘felt right’ to them, while others distrusted the same vaccines on an equally instinctive basis [28]. Such emotional intuition cannot always be anticipated or operationalized in advance, and therefore does not lend itself to structured preparedness strategies. For these reasons, the trust-building principle of *gut feeling* was set aside from the main comparative analysis.

After systematically examining each component of the five C framework, KP categorized the integration of the remaining public trust-building principles into three groups:

- Explicitly Present, which are principles that were directly mentioned, clearly articulated, and readily identifiable within the text of the five C framework.
- Implicitly Present, which are principles that were not overtly stated within the framework, but were inferred from its subtext, meaning that while the underlying concept was present, it was fragmented or not explicitly emphasized.
- Absent Principles, which are principles that were neither explicitly nor implicitly present within the five C framework.

To systematically determine whether trust-building principles were explicitly stated or implicitly embedded, the study employed a quality checklist originally developed for assessing the presence of such principles in European health data-sharing legislation [23]. Once the classification of themes was complete, any absent public trust-building principles were identified. These absent principles highlight areas where public trust-building principles could be further integrated into the five C framework to enhance public trust in pandemic and PHE preparedness and response. KP conducted the initial comparative analysis and categorization of explicitness, implicitness, and absence, while FG independently reviewed the coding. Any differences in interpretation were discussed between KP and FG until consensus was reached, ensuring consistency and reliability in the categorization process. This research was guided by the Standards for Reporting Qualitative Research (SRQR) checklist. This methodological approach ensured a structured and rigorous assessment of trust-building principles within the World Health Organization’s framework.

## Results

### Framework comparative analysis

#### Collaborative surveillance

*Active regulatory systems* and *security* are explicitly seen within the section; however, *net benefit* is implicitly deduced. The absent principles identified in this section were the themes of *familiarity*, *information quality*, *privacy*, and *time*.

#### Community protection

*Active regulatory systems*, *familiarity*, *information quality*, *net benefit*, and *time* are explicitly seen within the section. *Autonomy*, *certainty about the future*, and *respect* are all implicitly present within the text. There are no absent trust-building principles within this section.

#### Safe & scalable care

*Active regulatory systems* and *potential* are explicitly mentioned within the framework, while *certainty about the future, net benefit*, and *security* are implicitly present. The absent principle identified in this section is *time*.

#### Access to countermeasures

*Active regulatory systems*, *potential*, and *information quality* are explicitly mentioned within the framework, while *certainty about the future*, and *net benefit* are implicitly present. The absent principles identified in access to countermeasures were *anonymity* and *time*.

#### Emergency coordination

*Active regulatory systems* and *certainty about the future* are explicitly mentioned within the framework, while *net benefit* and *potential* are implicitly present. The absent principles identified in this section are *information quality* and *time*.

## Discussion

Incorporating public trust building principles into pandemic preparedness is essential for effective public health responses during the next pandemic. While not all trust-building principles that factor into public trust in the health system correlate to pandemic preparedness, there are four significant principles that are present in nearly all of the section in the World Health Organization’s five C framework; as well as significant gaps that need to be addressed in order to better integrate trust into pandemic preparedness and response.

### The dominant trust-building principles in the pandemic preparedness framework

When comparing the two frameworks (Table 3) there are many trust-building principles that are already evident within the five C framework, both explicitly and implicitly. *Active regulatory systems*, *certainty about the future*, and *net benefit* are consistent themes present across the framework. This is indicative of the nature and purpose of the five C framework.

**Table 3.** Trust-building themes present and absent within the World Health Organization’s five C framework, based on the public trust in the health system framework.

Five Cs	Trust Principles – Explicitly Present	Trust Principles – Implicitly Present	Absent Principles
Collaborative Surveillance	−Active Regulatory Systems −Security	−Net Benefit	−Familiarity −Information Quality −Privacy −Time
Community Protection	−Active Regulatory Systems −Familiarity −Information Quality −Net Benefit −Time	−Autonomy −Certainty about the Future −Respect	-N/A
Safe & Scalable Care	−Active Regulatory Systems −Potential	−Certainty about the Future −Net Benefit −Security	−Time
Access to Countermeasures	−Active Regulatory Systems −Potential −Information Quality	−Certainty about the Future −Net Benefit	−Anonymity −Time
Emergency Coordination	−Active Regulatory Systems −Certainty about the Future	−Potential −Net Benefit	−Information Quality −Time

The framework encompasses the core capacities of the International Health Regulations from 2005, a comprehensive legal framework outlining countries’ rights and responsibilities for managing public health events and emergencies that have the capability of crossing international borders. This highlights the importance of strong regulatory systems in the five C framework [4,29]. Therefore, weaving *active regulatory systems* into all aspects of the five C framework is a clear response to this issue and is expected to be seen.

*Certainty about the future* is also to be expected regularly in this framework as the concept itself concerns researchers and officials attempting to foresee possible risks in the future to the best of their capabilities, which is the main focus and objective of the five C framework [4,8]. *Certainty about the future* is linked to all Cs in the framework other than collaborative surveillance, which is appropriate. This is because surveillance is conducted when more information and intelligence is needed on a certain subject. As the very act of surveillance is predicated on trying to better understand what you are surveilling, being certain about the future is a product of surveillance, so it is inherently not possible to be certain about the future when conducting surveillance [30–32].

*Net benefit* is a fundamental component of both public trust and pandemic preparedness frameworks. In these contexts, *net benefit* reflects the concept of contributing positively to individuals or the collective system. People are more inclined to trust when they perceive personal benefits, for others or the system as a whole [8]. Similarly, in pandemic preparedness, ensuring readiness serves the best interests of society, aiming to achieve positive net health outcomes for all [4]. Due to this, *net benefit* is a consistent trust-building theme across the entire five C framework, whether explicitly or implicitly stated.

### Framework gaps and lingering issues

In the collaborative surveillance section, several trust principles were absent, including *familiarity*, *information quality*, *privacy*, and *time*. *Familiarity* must be present in regard to surveillance, as disease surveillance requires voluntary participation from the public in order to complete investigations and gather intelligence that can determine the spread of infection [33,34]. Yet, the public will be less willing to engage and actively participate in these surveillance operations if they do not have prior positive experiences with the organization(s) conducting the surveillance, such as the government [8,11,33,34]. Public trust is also influenced by *information quality*, as the public would be more willing to trust officials and organizations conducting disease surveillance if clear and transparent information is communicated to them in a timely and effective manner [11,21]. In addition, *privacy* must be considered when conducting disease surveillance, as if the public perceives that there is a risk to their private health information, they would lose trust in the process and would be more likely to refrain from participating in disease surveillance measures [8,33]. As for *time*, although collaborative surveillance relies on timely data collection and rapid information sharing, the framework does not address how sustained or accelerated surveillance activities are communicated to, experienced by, or perceived by the public, rendering time absent as a trust-building principle in this section [4,8]. These absences are particularly consequential for surveillance effectiveness, as collaborative surveillance relies heavily on voluntary public participation and sustained engagement. Without familiarity with surveillance actors, clear communication about data use, assurances of privacy protection, and attention to how surveillance activities are perceived when implemented rapidly or expanded during threats, surveillance systems may face reduced participation and weaker situational awareness when early detection is most critical [4,8,11,33,34].

For the section, community protection, all appropriate trust-building principles are present. Even so, while *autonomy* is implied within the section, individual autonomy is a controversial issue during a pandemic. As was seen during COVID-19, not all members of a society adhere to public health measures and argue that being forced to comply with these measures strips them of their autonomy [35,36]. Though this section states that there should be a ‘systematic approach to acceptance, adherence, and trust’ in order to promote adherence and minimize the negative consequences of public health measures during a PHE, there is no statement on how to maintain trust in those who are forced to comply to public health measures as a result of a PHE and therefore feel their autonomy is stripped away [4]. This must be addressed in the framework in order to increase the trust of these members of the public. In summation, the relatively comprehensive integration of trust-building principles in community protection is particularly important, as this domain involves highly visible, public-facing interventions where acceptance, adherence, and sustained cooperation are essential for effectiveness during a public health emergency [4,35,36].

In the section for safe and scalable care, the absent principle is *time*. While *time* is implicitly present through an emphasis on rapid scale-up, it remains a trust-relevant gap, as the framework does not address how accelerated care delivery may be perceived as rushed by the public. If these actions are believed to be rushed, they can cause a decrease of trust due to the notion that the intervention in question did not receive the proper check and balances (i.e. *time*) that should be given to said intervention [8,37,38]. In this domain, the absence of trust-relevant *time* considerations is especially consequential, as rapid expansion of healthcare capacity is highly visible to the public. When accelerated care delivery is perceived as rushed or insufficiently deliberated, concerns about quality and safety may undermine trust in health systems at the moment when reliance on those systems is greatest [8,37,38].

For access to countermeasures, the absent principles identified was *anonymity*. *Anonymity* was identified as a gap in the subsection for research and development, as there is no mention of ensuring data anonymity when conducting research. If the public is to trust and willingly participate in public health research, there must be explicit safeguards that ensure that any and all collected data remain anonymous [8,11]. *Time* is also considered an absent principle in the same way it is in safe and scalable care. It is explicitly mentioned within the section, but there should be an explicit statement discussing how actions would be taken by public health stakeholders to combat potential perceptions that quickly implemented PHE actions were unnecessarily and unsafely hastened [8,37,38]. These gaps are particularly salient in the context of access to countermeasures, where public trust is a prerequisite for participation in research, uptake of medical products, and adherence to deployment strategies. The absence of explicit safeguards for anonymity, combined with limited attention to how accelerated development timelines are perceived, risks reinforcing concerns about safety, oversight, and data protection that were widely observed during the COVID-19 vaccine rollout [8,11,37–39].

In the final section, emergency coordination, the absent principles identified are *information quality* and *time*. In relation to *information quality*, while this section provides comprehensive valuable ideas and recommendations for addressing gaps in response coordination at national, regional, and global levels, it does not propose strategies for effectively communicating these improvements to the public. The public’s criticism of the fragmented and inconsistent responses to COVID-19 highlights widespread awareness of the inadequacies in current coordination mechanisms for PHEs [4,40–42]. Merely enhancing these mechanisms and procedures is insufficient. It is equally crucial to transparently communicate the improvements and their outcomes clearly and effectively to the public. Such communication is essential to rebuild trust in public health stakeholders before the next pandemic arises [8,21,43–45]. As for *time*, although emergency coordination inherently requires rapid decision-making and swift mobilization of resources, the framework does not address how accelerated coordination processes are perceived by the public or how concerns about rushed or opaque decision-making might be mitigated. Evidence from the COVID-19 response suggests that delays, abrupt policy shifts, and poorly explained timing of decisions undermined public trust in coordinated governance responses, underscoring the importance of addressing temporal perceptions alongside operational speed [4,40–42,46]. In emergency coordination, the absence of both *information quality* and trust-relevant *time* considerations is particularly problematic, as coordination failures are often experienced by the public through fragmented messaging, abrupt policy shifts, and poorly explained timing of decisions. Without transparent communication and attention to how rapid coordination is perceived, improvements in technical coordination alone may fail to translate into restored public confidence during crises [4,39–45]

While each of the five Cs revealed distinct limitations, some trust principles were more central to the gaps than others. Overall, *time* was the most consistently absent principle, while *information quality, familiarity*, and *privacy* were more limited, but notable, omissions. Taken together, these gaps highlight the most prominent areas in which the five Cs could more explicitly support public trust. However, the uneven treatment of *time* across the framework warrants separate consideration, as it cuts across multiple domains and presents distinct challenges that are not captured by a section-by-section analysis. While temporal considerations appear across multiple sections, they are primarily framed in procedural terms and are unevenly incorporated from a trust-building perspective. Accordingly, time was categorized as explicitly present only where temporal considerations extended beyond implementation speed to include public engagement and acceptance, and as absent where perception management of rapid action was not addressed. Its presence or absence depends on how it is viewed. For example, in a PHE, quickly scaling up essential public health services and medical care is imperative. Therefore, the five C framework explicitly emphasizes the importance of timely and appropriate action. However, it does not address the risk that the public may perceive this rapid response as rushed. Even if the process was thorough, a perception of haste can lead to a reduction of public trust. People are less likely to trust those in charge of actionable decision-making and engage with an intervention if they believe said intervention was not given enough time and care [8,37,38]. Utilizing COVID-19 vaccination as an example, the rapid development of COVID-19 vaccines, while necessary, led to public concerns about their safety. This perception has been observed across pandemic contexts, health systems, and socioeconomic settings, and has contributed to increased vaccine hesitancy and lower vaccine uptake [39,47–49].

Therefore, while timeliness is frequently emphasized within the five C framework, the extent to which time functions as a trust-building principle varies across sections depending on whether public perceptions of decision-making speed are addressed. Further complicating the issue, while PHE response needs to be timely and appropriately swift in order to be effective, higher trust has also been found to be associated with slower policy response times, yet being too slow can lead to a lowering of trust as well [4,46]. Threading this needle is extremely difficult and is indicative of the whole issue of *time* in the context of trust during a pandemic or other type of PHE. Though, because of its difficult nature, it should be one of the top priority concerns for public health stakeholders to address before the next pandemic or large-scale PHE.

### How to strengthen public trust-building principles in the World Health Organization’s five C framework

To ensure effective and comprehensive pandemic preparedness, public health stakeholders must exercise both care and authority in addressing existing trust gaps in pandemic preparedness frameworks. Trust-building principles must be thoughtfully integrated into pandemic preparedness frameworks to bolster their trustworthiness and effectiveness [21]. In the case of the five Cs, a trustworthy framework, one that incorporates trust-building and/or trust-strengthening principles at all appropriate junctions, should serve as the foundation for World Health Organization member nations’ preparedness strategies. By doing so, democratic nations can establish effective preparedness measures and safeguards, ensuring they are equipped to respond to future pandemics or PHEs with efficiency, effectiveness, and public support. Trust is not just a supplementary component but a cornerstone of preparedness. Without it, even the most meticulously designed frameworks may fail to achieve their intended outcomes in guiding and encouraging proper preparation for the next pandemic or PHE [21,48]. As well, incorporating trust into pandemic preparedness frameworks must take into account the needs and concerns of each country’s population, necessitating tailored approaches that will be specific to each country’s pandemic preparedness framework [21]. No matter which framework it is though, from a community plan to an international one, beginning to address the trust gaps now during the current ‘cold period’ before another pandemic or PHE arises is imperative.

To address the gaps formed by trust-building principles that were absent in the World Health Organization’s five C framework, the principles of *familiarity*, *information quality*, *privacy*, *autonomy*, *anonymity*, and certain aspects of *time* need to be emphasized in the respective Cs in which they are lacking. By explicitly embedding these principles within the five C framework, the framework can more comprehensively and effectively address the issues of trust currently surrounding pandemic preparedness measures, which we see are fueled consistently by a lack of accurate information, inconsistent communication, and social and individual perceptions and tensions [12,14,21]. Having clear and explicit trust principles within the framework, guided by tools, positive examples, research, and experts in the field, will foster trust in public health authorities and institutions, and will aid in both the dissemination of accurate, evidence-based information and in improving community engagement and acceptability of pandemic preparedness measures and policies [8,21].

### Policy and practice implications

Public health professionals have a duty to proactively safeguard population health by preventing and mitigating risks before they escalate. The lessons of past crises underscore not only the consequences of inadequate preparedness, but also the dangers of insufficient public trust in essential PHE interventions [1,2,49,50]. Building and maintaining trust must therefore be treated as a core component of preparedness, rather than a peripheral concern.

These recommendations could be operationalized by embedding public trust-building principles into existing evaluation and planning processes used by World Health Organization member states. For example, within the Joint External Evaluation process, indicators could be expanded to assess the transparency, clarity, and inclusiveness of communication strategies, or to evaluate whether public perceptions of autonomy and privacy are systematically considered in preparedness planning [51]. Such an approach could include a Joint External Evaluation indicator assessing whether countries have established formal mechanisms for two-way communication with communities regarding surveillance activities, evaluating not only whether information is disseminated but also whether public concerns related to privacy, data use, and governance are systematically collected, documented, and addressed within surveillance protocols. Similarly, National Action Plans for Health Security could incorporate explicit benchmarks requiring governments to demonstrate how familiarity, information quality, and privacy are integrated into preparedness measures [52]. In practice, this would mean aligning technical capacities with mechanisms for public engagement, communication, and oversight, ensuring that public trust is treated not as a secondary outcome but as a measurable component of preparedness itself.

Thusly, incorporating public trust-building principles into preparedness frameworks is an important first step, but it should be a foundational priority rather than an afterthought. The guiding tenets of these frameworks must be embedded into policies, actionable operational strategies, and the foundations of national healthcare infrastructures [8,21]. This requires not only technical integration but also political commitment and resource allocation, ensuring that public trust is institutionalized across levels of governance. Without this, public trust risks remaining a rhetorical goal rather than a practical element of preparedness, limiting the effectiveness of both international and national pandemic preparedness strategies, along with the Joint External Evaluation process and National Action Plans for Health Security.

### Strengths, limitations, and reflexivity

One limitation of this study is that pandemic preparedness, as success or failure is usually determined toward the end or in the aftermath of a pandemic, making the assessments in this article hard to demonstrate until the next pandemic event. A second limitation is this study’s reliance on a small-scale preliminary literature search and a comparative analysis rather than empirical data collection or case studies to validate the proposed recommendations. While comparative analysis is a useful first step for identifying conceptual gaps, it cannot substitute for empirical testing in applied settings and it does not account for contextual differences in how frameworks are implemented across countries, nor does it test whether the absence of specific principles necessarily translates into weaknesses in preparedness outcomes. The classification of trust-building principles as explicit, implicit, or absent necessarily involved a degree of interpretive judgment. Although dual coding and consensus discussions were used to enhance analytical rigor, formal inter-rater reliability statistics were not calculated. As a result, some variability in categorization cannot be fully ruled out. A further limitation of this study is its focus on the World Health Organization’s five C framework. Although selected for its global applicability, it is not the only preparedness and response framework available. As mentioned earlier, other frameworks, such as those created by the US and European Centers for Disease Control, also guide how member states assess and strengthen preparedness. By centering the five C framework, this analysis may overlook insights offered by these alternative approaches, particularly in relation to technical capacities and legal obligations.

However, one strength of this research is that both the five C framework and the public trust in the health system framework are established frameworks and based on years of knowledge and experience. As well, another strength of this study is its applicability to both global and national public health pandemic preparedness frameworks, due to the utilization of the World Health Organization’s framework, and to existing research on the topic of public trust incorporation into pandemic preparedness, such as the work of Bollyky and Petersen [21]. This comparative framework analysis also represents an important foundation for future empirical testing of the results and recommendations provided by this study, as framework analysis is an appropriate first step that translates abstract trust principles into specific, evaluable components of pandemic preparedness. By establishing this groundwork, subsequent research can more effectively examine the practical application and impact of these recommendations.

As the primary researchers, KP and FG bring a combined experience of over 12 years in trust-related research, which may introduce certain preconceived notions about public trust. We acknowledge that FG’s involvement in the development of the public trust framework may have predisposed the research team toward identifying gaps in the preparedness framework. To mitigate this risk, we applied a structured quality assessment checklist adapted from prior research, with categorization criteria defined a priori rather than developed post hoc. In addition, review of the findings by VvW provided an external perspective on interpretation, supporting reflexivity and analytic balance. Additionally, as KP and VvW were not involved in the development of the public trust in the health system framework, their input offered a more neutral lens through which to assess the findings. This helped to counterbalance any potential biases held by FG in relation to the framework, thereby strengthening the credibility and objectivity of the analysis.

## Conclusions

While the World Health Organization’s framework is thorough and contains many public trust-building principles in place, important gaps remain that need to be addressed in the interim before the next pandemic. Trust-building is a long and complex process, and it becomes far more difficult once a crisis is underway. To avoid repeating the mistakes of the COVID-19 response, efforts to strengthen trust must begin during the inter-pandemic period, when there is greater opportunity to build familiarity, improve information quality, and address privacy concerns. Embedding these principles into routine preparedness mechanisms, such as Joint External Evaluations and National Action Plans for Health Security, would ensure that trust-building is sustained and institutionalized rather than improvised during emergencies. Only by prioritizing trust now can public health systems be fully prepared for the challenges of the next pandemic, and there is no better time to start than the present.

## Supplementary Material

Pub Trust in Pan Prep_SRQR Checklist_Appendix A.docx

## Data Availability

The authors confirm that the data supporting the findings of this study are available within the article and its supplementary materials.

## Data deposition

https://www.zora.uzh.ch/
